# Profiling of VEGFs and VEGFRs as Prognostic Factors in Soft Tissue Sarcoma: VEGFR-3 Is an Independent Predictor of Poor Prognosis

**DOI:** 10.1371/journal.pone.0015368

**Published:** 2010-12-14

**Authors:** Thomas K. Kilvaer, Andrej Valkov, Sveinung Sorbye, Eivind Smeland, Roy M. Bremnes, Lill-Tove Busund, Tom Donnem

**Affiliations:** 1 Institute of Medical Biology, University of Tromso, Tromso, Norway; 2 Department of Clinical Pathology, University Hospital of North Norway, Tromso, Norway; 3 Department of Oncology, University Hospital of North Norway, Tromso, Norway; 4 Institute of Clinical Medicine, University of Tromso, Tromso, Norway; National Cancer Institute, United States of America

## Abstract

**Background:**

In non-gastrointestinal stromal tumor soft tissue sarcoma (non-GIST STS) optimal treatment is surgery with wide resection margins. Vascular endothelial growth factors (VEGFs) and receptors (VEGFRs) are known to be key players in the initiation of angiogenesis and lymphangiogenesis. This study investigates the prognostic impact of VEGFs and VEGFRs in non-GIST STS with wide and non-wide resection margins.

**Methods:**

Tumor samples from 249 patients with non-GIST STS were obtained and tissue microarrays were constructed for each specimen. Immunohistochemistry was used to evaluate the expressions of VEGF-A, -C and -D and VEGFR-1, -2 and -3.

**Results:**

In the univariate analyses, VEGF-A (P = 0.040) in the total material, and VEGF-A (P = 0.018), VEGF-C (P = 0.025) and VEGFR-3 (P = 0.027) in the subgroup with wide resection margins, were significant negative prognostic indicators of disease-specific survival (DSS). In the multivariate analysis, high expression of VEGFR-3 (P = 0.042, HR = 1.907, 95% CI 1.024-3.549) was an independent significant negative prognostic marker for DSS among patients with wide resection margins.

**Conclusion:**

VEGFR-3 is a strong and independent negative prognostic marker for non-GIST STSs with wide resection margins.

## Introduction

Soft tissue sarcomas (STS) originate from the mesenchymal lineage, and thus share a similar ancestry [1]. Despite the fact that the STS group of tumors cover over 50 different histological entities, the occurrence of these tumors amounts to only 0.5% of the annual cancer incidence [1], [2]. The STSs are among the most aggressive cancer types [2] with a lethality of 40–50%. About 10.000 new cases and 4.000 related deaths were registered in the US in 2009 [2].

Classically STSs have been treated as a single group. This is mainly because the low incidence makes it difficult to conduct decently powered studies on the individual histological entities. With the emerging knowledge of cellular processes and the increasing knowledge about common and uncommon genetic translocations the last decade, it is now clear that the picture might be more intricate. For instance, Ewing family tumors, synovial sarcoma, rhabdomyosarcoma, dermatofibrosarcoma protuberens and others have distinct genetic translocations [3], [4], [5]. However, the genetic translocations specific for the histological entities have few implications for treatment options. Therefore it is still adequate to group the remaining STSs together under the proposed name of non-gastrointestinal stromal tumor STS (non-GIST STS), although this might change in the future [4].

The main treatment of sarcomas is surgical resection, and wide resection margins are considered one of the most important prognostic factors [6]. However, a considerable variability in prognosis has been observed for subsets of patients with wide resection margins. Consequently, the clinical incorporation of predictive and prognostic molecular biomarkers together with traditional clinical prognostic factors will be pivotal for future management of patients within this large subgroup.

Angiogenesis inhibitors provide a new and exciting therapeutic option for patients with STS [7]. However, the angiogenesis pathway in STS needs to be further examined to improve the treatment strategy [7].The vascular endothelial growth factors (VEGFs) and their receptors (VEGFRs) are well known targets in antiangiogenic treatment. The VEGF super-family consist of six ligands, placental growth factor (PlG), VEGF-A, -B, -C, -D and -E, and three receptors, VEGFR-1, -2 and -3. VEGFR-1 binds PlG and VEGF-A and -B, VEGFR-2 binds VEGF-A, -C and -D and VEGFR-3 binds VEGF-C and -D [8]. VEGF-A signaling through VEGFR-2 is considered the major angiogenic pathway, leading endothelial cells (ECs) to proliferate, sprout and form tubes. VEGFR-1 signaling has been implicated in regulating VEGFR-2 mediated angiogenesis [9]. VEGF-C and VEGF-D have been shown to induce lymphangiogenesis through VEGFR-3 [8], [10]. The latter has also been implicated in controlling angiogenic sprouting [11].

High levels of VEGF-A in tumors and blood samples from STS patients have previously been associated with higher tumor grade, increased tendency to metastasis, reduced response to treatment, lower overall survival (OS) and increased risk of recurrence [12], [13], [14], [15], [16], [17]. In angiosarcomas, however, high expression of VEGFR-2 has been associated with longer OS [18]. VEGF-C and VEGFR-3 overexpression has also been reported in STSs [19].

In this study, the aim was to assess the prognostic impact of VEGF-A, -C, -D and VEGFR-1. -2 and -3 in non-GIST STS patients with wide and non-wide resection margins.

## Methods

### Patients and Clinical Samples

Primary tumor tissue from anonymized patients diagnosed with non-GIST STS at the University Hospital of North-Norway and the Hospitals of Arkhangelsk county, Russia, from 1973 through 2006, were collected. In total 496 patients were registered from the hospital databases. Of these 247, patients were excluded from the study due to: missing clinical data (n = 86) or inadequate paraffin-embedded fixed tissue blocks (n = 161). Thus 249 patients with complete medical records and adequate paraffin-embedded tissue blocks were eligible.

This report includes follow-up data as of September 2009. The median follow-up was 37.6 (range 0.1–391.7) months. Complete demographic and clinical data were collected retrospectively. Formalin-fixed and paraffin-embedded tumor specimens were obtained from the archives of the Departments of Pathology at the University Hospital of North-Norway and the Hospitals of Arkhangelsk County. The tumors were graded according to the French Fédération Nationale des centres de Lutte Contre le Cancer (FNCLCC) system and histologically sub typed according to the World Health Organization guidelines [1], [20]. Wide resection margins were defined as wide local resection with free microscopic margins or amputation of the affected limb or organ. Non-wide resection margins were defined as marginal or intralesional resection margins, or no surgery.

### Microarray construction

All sarcomas were histologically reviewed by two trained pathologists (S. Sorbye and A. Valkov) and the most representative areas of tumor cells (neoplastic mesenchymal cells) were carefully selected and marked on the hematoxylin and eosin (H/E) slide and sampled for the tissue microarray (TMA) blocks. The TMAs were assembled using a tissue-arraying instrument (Beecher Instruments, Silver Springs, MD). The Detailed methodology has been previously reported [21]. Briefly, we used a 0.6 mm diameter stylet, and the study specimens were routinely sampled with four replicate core samples from different areas of neoplastic tissue. Normal tissue from the patients was used as staining control.

To include all core samples, 12 TMA blocks were constructed. Multiple 5-µm sections were cut with a Micron microtome (HM355S) and stained by specific antibodies for immunohistochemistry (IHC) analysis.

### Immunohistochemistry

The applied antibodies were subjected to in-house validation by the manufacturer for immunohistochemical analysis on paraffin-embedded material. The antibodies used in the study were as follows: VEGF-A (1∶10, rabbit polyclonal; RB-1678; Neomarkers), VEGF-C (1∶25, rabbit polyclonal; 18-2255; Zymed Laboratories), VEGF-D (1∶40, mouse monoclonal; MAB286; R&D Systems), VEGFR-1 (1∶10, rabbit polyclonal; RB-1527; Neomarkers), VEGFR-2 (1∶25, rabbit polyclonal; RB-9239; Neomarkers), and VEGFR-3 (1∶10, rabbit polyclonal; Sc-321; Santa Cruz Biotechnology).

Sections were deparaffinized with xylene and rehydrated with ethanol. Antigen retrieval was done by placing the specimen in 0.01 mol/L of citrate buffer at pH 6.0 and exposed to repeated (twice) microwave heating of 10 min (except VEGFR-3, twice for 5 min) at 450 W. VEGF-D was heated for 45 min in a water bath in 0.01 mol/L of citrate buffer. The DAKO EnVision+ System-HRP kit (diaminobenzidine) was used for endogen peroxidase blocking. As negative staining controls, the primary antibodies were replaced with the primary antibody diluents. Primary antibodies were incubated for 30 min in room temperature (except VEGFR-3, 20 min, and VEGF-D, overnight at 4°C). The DAKO EnVision+ System-HRP kit (diaminobenzidine) was used to visualize the antigens. This was followed by the application of liquid diaminobenzidine and substrate-chromogen, yielding a brown reaction product at the site of the target antigen. Finally, all slides were counterstained with hematoxylin to visualize the nuclei. For each antibody, included negative staining controls, all TMA staining were done in a single experiment.

### Scoring of immunohistochemistry

The ARIOL imaging system (Genetix, San Jose, CA) was used to scan the slides of antibody staining of the TMAs. The slides were loaded in the automated slide loader (Applied Imaging SL 50) and the specimens were scanned at low resolution (1.25×) and high resolution (20×) using the Olympus BX 61 microscope with an automated platform (Prior). Representative and viable tissue sections were scored manually on computer screen semi quantitatively for cytoplasmic staining. The dominant staining intensity was scored as: 0 =  negative; 1 =  weak; 2 =  intermediate; 3 =  strong. All samples were anonymized and independently scored by two trained pathologists (A. Valkov and S. Sorbye). When assessing a variable for a given core, the observers were blinded to the scores of the other variables and to outcome. Mean score for duplicate cores from each individual was calculated separately.

**Figure 1:**
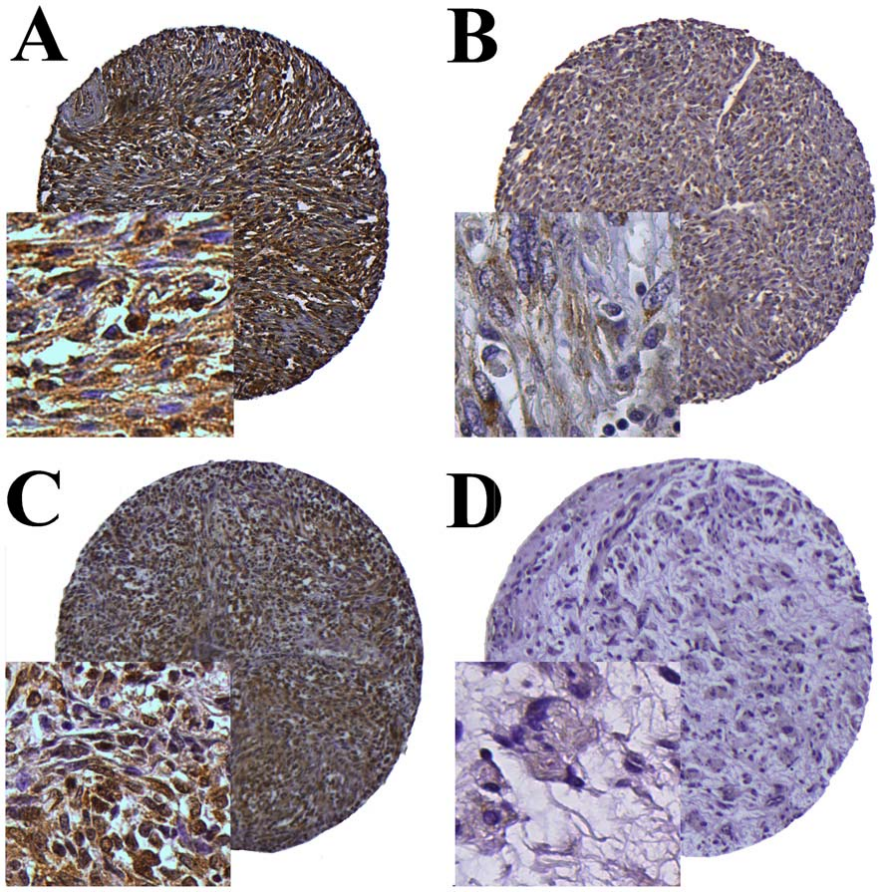
IHC analysis of TMA of non-GIST STSs representing different scores for tumor cell VEGF-C and VEGFR-3. (A) Tumor cell VEGF-C high score in leiomyosarcoma; (B) Tumor cell VEGF-C low score in leiomyosarcoma; (C) Tumor cell VEGFR-3 high score in undifferentiated pleomorphic sarcoma; (D) Tumor cell VEGFR-3 low score in liposarcoma. Abbreviations: IHC, immunohistochemistry; non-GIST STS, non-gastrointestinal stromal tumor soft-tissue sarcoma TMA, tissue microarray; VEGF, vascular endothelial growth factor; VEGFR, vascular endothelial growth factor receptor.

High expression in tumor cells was defined as score ≥1.5 (VEGF-A, VEGF-D, VEGFR-1-3) and ≥1 (VEGF-C) (Fig. 1).

### Statistical Methods

All statistical analyses were done using the statistical package SPSS (Chicago, IL), version 16. The IHC scores from each observer were compared for interobserver reliability by use of a two-way random effect model with absolute agreement definition. The intraclass correlation coefficient (reliability coefficient) was obtained from these results. The Chi-square test and Fishers Exact test were used to examine the association between molecular marker expression and various clinicopathological parameters. Univariate analyses were done using the Kaplan-Meier method, and statistical significance between survival curves was assessed by the log rank test. DSS was determined from the date of diagnosis to the time of cancer related death. To assess the independent value of different pretreatment variables on survival, in the presence of other variables, multivariate analyses were carried out using the Cox proportional hazards model. Only variables of significant value from the univariate analyses were entered into the Cox regression analyses. Probability for stepwise entry and removal was set at .05 and .10, respectively. The significance level used for all statistical tests was P<0.05.

### Ethical clearance

The National Data Inspection Board and The Regional (Northern Norway) Committee for Research Ethics approved the study.

## Results

### Clinopathological Variables

The clinopathological variables are summarized in Table 1. The median age was 59 (range 0–91) years, 56% were female, 167 patients were Norwegian and 82 Russian. The Non-GIST STSs comprised 249 tumors including angiosarcoma (n = 13), fibrosarcoma (n = 20), leiomyosarcoma (n = 64), liposarcoma (n = 34), undifferentiated pleomorphic sarcoma (n = 58), neurofibrosarcoma/malignant peripheral nerve sheath tumor (MPNST, n = 11), rhabdomyosarcoma (n = 16), synovial sarcoma (n = 16) and unspecified sarcoma (n = 17). The tumor origins were distributed as follows: 36% extremities, 19% trunk, 15% retroperitoneal, 7% head/neck and 23% visceral. Of 228 patients who underwent surgery, 53% received surgery alone, 24% surgery and radiotherapy, 18% surgery and chemotherapy and 6% surgery, radiotherapy and chemotherapy. Besides, 21 patients did not undergo surgery due to inoperable tumor (n = 11), high age/other serious disease (n = 5), STS confirmed at autopsy (n = 3) and patient refusal (n = 2). Of these unresected patients, seven patients received chemotherapy and/or radiotherapy, whereas 14 patients received no anticancer therapy.

**Table 1:** Prognostic relevance of clinicopathological variables for disease-specific survival in 249 non-gastrointestinal stromal tumor soft-tissue sarcomas (univariate analyses, log rank test).

Characteristics	Patients (n)	Patients (%)	Median survival (months)	5-Year survival (%)	P
**Age**					
≤ 20 years	20	8	15	40	0.126
21–60 years	113	45	68	52	
>60 years	116	47	30	40	
**Gender**					
Male	110	44	41	46	0.390
Female	139	56	45	45	
**Patient nationality**					
Norwegian	167	67	63	51	0.011
Russian	82	33	22	34	
**Histological entity**					
UndifferentiatedPleomorphic sarcoma	58	23	54	47	0.001
Leiomyosarcoma	64	26	48	48	
Liposarcoma	34	14	NR	67	
Fibrosarcoma	20	8	44	50	
Angiosarcoma	13	5	10	31	
Rhabdomyosarcoma	16	6	17	38	
MPNST	11	4	49	45	
Synovial sarcoma	16	6	31	29	
Sarcoma NOS	17	7	9	18	
**Tumor localization**					
Extremities	89	36	100	53	0.348
Trunk	47	29	32	44	
Retroperitoneum	37	25	25	38	
Head/Neck	18	7	15	41	
Visceral	58	23	30	42	
**Tumor size**					
≤5 cm	74	30	127	57	0.027
5–10 cm	91	37	44	45	
>10 cm	81	32	28	37	
Missing	3	1			
**Malignancy grade**					
1	61	25	NR	74	<0.001
2	98	39	41	45	
3	90	36	16	26	
**Tumor depth**					
Superficial	17	7	NR	93	<0.001
Deep	232	93	36	42	
**Metastasis at diagnosis**					
No	206	83	76	53	<0.001
Yes	43	17	10	10	
**Surgery**					
Yes	228	92	59	50	<0.001
No	21	8	5	0	
**Resection margins**					
Wide	108	43	NR	62	<0.001
Non-wide/no surgery	141	57	21	33	
**Chemotherapy**					
No	191	77	52	47	0.424
Yes	58	23	29	40	
**Radiotherapy**					
No	176	71	48	46	0.590
Yes	73	29	38	43	

Abbreviations: NR, not reached; MPNST, malignant peripheral nerve sheath tumor; NOS, not otherwise specified.

### Interobserver variability

Interobserver scoring agreement was tested for one ligand (VEGF-C) and one receptor (VEGFR-3). The intraclass correlation coefficients were 0.810 for VEGF-C (P<0.001) and 0.834 for VEGFR-3 (P<0.001) indicating good reproducibility between the two investigating pathologists.

### Expression of VEGFs/VEGFRs and their Correlations

VEGF/VEGFR expression was observed in the cytoplasm of tumor cells. For the ligand and receptor expressions we found the following correlation with malignancy grade: High VEGF-A expression, grade 1: 29%, grade 2: 48%, grade 3: 56% (P = 0.005); High VEGF-C expression, grade 1: 24%, grade 2: 41%, grade 3: 45% (P = 0.032); High VEGFR-1 expression, grade 1: 27%, grade 2: 36%, grade 3: 48% (P = 0.034); High VEGFR-2 expression, grade 1: 12%, grade 2: 27%, grade 3: 39% (P = 0.001).

### Univariate Analyses

Table 1 summarizes the prognostic impact of the clinicopathological variables in the total material. In the univariate analyses, patient nationality (P = 0.011), histological entity (P = 0.001), tumor size (P = 0.027), malignancy grade (P<0.001), tumor depth (P<0.001), metastasis at diagnosis (P<0.001), surgery (P<0.001) and surgical margins (P<0.001) were all significant prognostic indicators for DSS. In the subgroup with wide resection margins (n = 108), patient nationality (P<0.001), malignancy grade (P<0.001), tumor depth (P = 0.009) and metastasis at diagnosis (P<0.001) were prognostic indicators of DSS. In the subgroup with non-wide resection margins (n = 141), malignancy grade (P<0.001), surgery (P<0.001), metastasis at time of diagnosis (P<0.001) and histological entity (P = 0.005) were prognostic indicators of DSS.

**Figure 2:**
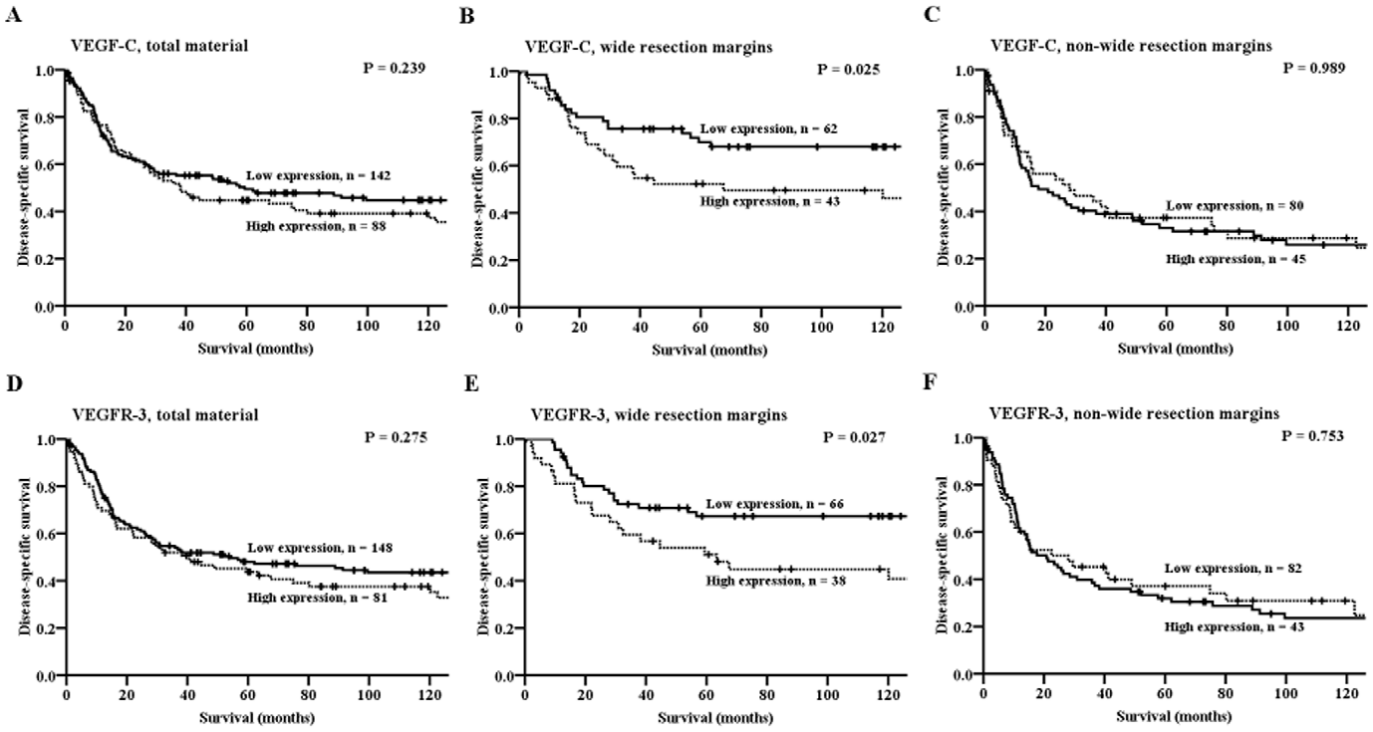
Disease-specific survival curves for VEGF-C and VEGFR-3 in the total material and in the group with wide and non-wide resection margins. (A) VEGF-C, total material; (B) VEGF-C, wide resection margins; (C) VEGF-C, non-wide resection margins; (D) VEGFR-3, total material; (E) VEGFR-3, wide resection margins; (F) VEGFR-3, non-wide resection margins. Abbreviations: VEGF, vascular endothelial growth factor; VEGFR, vascular endothelial growth factor receptor.

The influence on DSS by the VEGFs and VEGFRs are given in Table 2 and Figure 2 (VEGF-C and VEGFR-3). In the total material, VEGF-A expression (P = 0.040) was a significant negative prognostic indicator of DSS. In the subgroup with wide resection margins, VEGF-A (P = 0.018), VEGF-C (P = 0.025) and VEGFR-3 (P = 0.027) expressions were significant negative prognostic indicators of DSS. In the subgroup with non-wide resection margins, neither the VEGFs nor VEGFRs were indicators of DSS.

**Table 2:** Tumor expression of VEGFs and VEGFRs and their prognostic relevance for disease-specific survival in patients with non-gastrointestinal soft-tissue sarcomas in the total material (univariate analyses; log-rank test, N = 249) and in subgroups with wide and non-wide resection margins (univariate analyses; log-rank test, N = 108 and 141 respectively).

	Overall material					Wide resection margins					Non-wide resection margins					
Marker expression	Patients(n)	Patients(%)	Median survival(months)	5-Year survival(%)	P	Patients(n)	Patients(%)	Median survival(months)	5-Year survival(%)	P	Patients(n)	Patients(%)	Median survival(months)	5-Year survival(%)	P	
**VEGF A**																
Low	127	51	59	50	0.040	57	53	NR	69	0.018	70	50	21	34	0.508	
High	109	44	31	42		48	44	63	52		61	43	21	34		
Missing	13	5				3	3				10	7				
**VEGF C**																
Low	142	57	59	49	0.239	62	57	NR	70	0.025	80	57	18	33	0.989	
High	88	35	38	45		43	40	68	52		45	32	28	37		
Missing	19	8				3	3				16	11				
**VEGF D**																
Low	157	63	57	48	0.276	64	59	NR	64	0.267	93	66	23	37	0.169	
High	83	33	36	42		43	40	120	57		40	28	11	25		
Missing	9	4				1	1				8	6				
**VEGFR 1**																
Low	145	58	57	48	0.262	66	61	NR	64	0.110	79	56	23	34	0.963	
High	89	36	41	46		40	37	120	58		49	35	21	36		
Missing	15	6				2	2				13	9				
**VEGFR 2**																
Low	164	66	57	48	0.246	80	74	NR	65	0.135	84	60	18	31	0.689	
High	63	25	31	44		24	22	68	52		39	28	26	38		
Missing	22	9				4	4				18	13				
**VEGFR 3**																
Low	148	59	54	48	0.275	66	61	NR	67	0.027	82	58	21	32	0.753	
High	81	33	41	44		38	35	63	51		43	31	23	37		
Missing	20	8				34	4				16	11				

Abbreviations: NR, not reached.

### Multivariate Cox Proportional Hazards Analysis

Results of the multivariate analyses are presented in Tables 3 and 4. In the total material, tumor depth (P = 0.046), tumor size (P = 0.045), high malignancy grade (P<0.001), lack of surgery (P<0.001), non-wide resection margins (P = 0.004) and metastasis at diagnosis (P<0.001), but none of the angiogenic markers, were significant independent prognostic indicators of DSS (Table 3). In the wide resection margins group, Russian nationality (P = 0.013), high malignancy grade (P = 0.009), metastasis at diagnosis (P = 0.007) and high VEGFR-3 expression (P = 0.042, HR = 1.907, 95% CI 1.024-3.549) were significant independent prognostic indicators for reduced DSS (Table 4). In the group with non-wide resection margins, high malignancy grade (P<0.001), lack of surgery (P<0.001) and metastasis at time of diagnosis (P<0.001) were independent prognostic indicators of poor DSS.

**Table 3:** Results of the Cox regression analysis of the total material.

Factor	Hazard Ratio	95% CI	P
**Tumor depth**			
Superficial	1.000		
Deep	7.541	1.040–54.661	0.046
**Tumor size**			0.045*
≤5 cm	1.000		
5–10 cm	1.420	0.895–2.252	0.136
>10 cm	1.858	1.140–3.030	0.013
**Malignancy grade**			<0.001*
1	1.000		
2	2.892	1.660–5.040	<0.001
3	4.192	2.421–7.259	<0.001
**Surgery**			
Yes	1.000		
No	8.426	4.311–16.469	<0.001
**Resection margins**			
Wide	1.000		
Non-wide	1.785	1.209–2.637	0.004
**Metastasis at time of diagnosis**			
No	1.000		
Yes	2.551	1.672–3.893	<0.001

*Overall significance as a prognostic factor.

**Table 4:** Results of the Cox regression analysis among patients with wide resection margins.

Factor	Hazard Ratio	95% CI	P
**Patient nationality**			
Norwegian	1.000		
Russian	2.257	1.186–4.295	0.013
**Malignancy grade**			0.009*
1	1.000		
2	3.672	1.200–11.240	0.023
3	5.484	1.828–16.447	0.002
**Metastasis at time of diagnosis**			
No	1.000		
Yes	2.900	1.332–6.315	0.007
**VEGFR-3**			
Low	1.000		
High	1.907	1.024–3.549	0.042

*Overall significance as a prognostic factor.

## Discussion

In this study we observed that high expression of VEGFR-3 was a significant independent negative prognostic indicator of DSS in non-GIST STS patients with wide resection margins. Although there have been prior evaluations of the VEGF axis in STSs, these have primarily been focused on VEGF-A. Herein, we have presented a large-scale study of the prognostic impact of VEGF-A, -C and -D and VEGFR-1-3 in non-GIST STS patients. To our knowledge, this is the first evaluation of these pathways according to resection margins.

The major weakness of this study, normally seen in sarcoma studies in general, is the heterogeneity of the sarcoma population. Even with a relatively large sample cohort with regard to non-GIST STSs, the numbers are too small to do meaningful explorations according to histological subgroups, at least with respect to multivariate analysis.

Wide resection margins have been demonstrated to give the best overall survival, with more modest results for marginal and particularly intralesional resections [6]. Despite wide resection margins 40% of patients in our population succumbed to their sarcoma within five years. Identification of prognostic markers within this group of patients is therefore of great interest.

This is the first report of VEGFR-3 expression being an independent negative prognostic marker in non-GIST with wide resection margins. VEGFR-3 is a tyrosine-kinase receptor that is activated by VEGF-C and -D. The VEGFR-3/VEGF-C/-D system is considered the main pathway responsible for developing lymphatic vessels [8]. During the organogenesis, VEGFR-3 is expressed in all endothelia, but as the organism matures the expression has been associated mainly with lymphangiogenesis [22]. In a small series of 32 STSs, Friedrichs et al. found that around 50% of the tumors contained confirmable lymphatic vessels and expressed VEGFR-3 and VEGF-C [19]. In contrast, recent data have shown that VEGFR-3 is expressed in the lamellopodia of lead-cells in angiogenic sprouts, indicating that VEGFR-3 may play an important role also in angiogenesis [11]. This has been further supported by the fact that co-administration of VEGFR-2 and VEGFR-3 antibodies lead to a more extensive suppression of angiogenesis than VEGFR-2 antibodies alone [11]. Through Folkman's work on angiogenesis we know that without blood-supply a tumor cannot grow beyond 1–2 mm^3^ in size [23]. This means that the angiogenic capabilities of VEGFR-3 may be driving tumor angiogenesis and ultimately tumor development in non-GIST STS patients. As the vascular and not the lymphatic system is the principal metastatic pathway in non-GIST STSs, it is natural to assume that increased angiogenesis will augment the risk for metastasis development [24]. Increased vascularity will also lead to increased interstitial fluid pressure (IFP), which inhibits drug delivery to the tumor [25]. Since VEGFR-3 is a strong lymphangiogenic factor, one could assume a worse DSS mediated by high expression levels of VEGFR-3 was due to increased lymphangiogenesis and subsequent lymph node metastasis, although this is rare for sarcomas [24], [26]. VEGFR-3 may also function as a transducer of survival signals through autocrine pathways with tumor-derived VEGF-C or -D or autoactivation of the receptor itself [8].

In the presented population with wide resection margins, tumor VEGF-C expression was a significant negative prognostic marker for DSS. To our knowledge, only one small study has previously reported on this relationship in STSs. In 45 patients with undifferentiated pleomorphic sarcoma (previously malignant fibrous histiocytoma, MFH) and neurogenic sarcoma, Hoffmann et al. concluded surprisingly that high expression of VEGF-C mRNA led to a longer overall survival [27]. This is inconsistent with our findings and may be explained by sampling variation or lacking translation of mRNA to protein in the tumors. VEGF-C can interact with both VEGFR-2 and VEGFR-3, leading to migration of ECs and increased capillary permeability [8], [9]. These effects are thought to be mediated through VEGFR-2 in vascular ECs and through VEGFR-3 in lymphatic ECs [8], [9]. In tumors this will lead to angiogenesis, lymphangiogenesis and increased IFP, which promote tumor sustenance, progression, metastasis and resistance to cytotoxic therapy.

We found VEGF-A expression in tumor tissue to be a significant negative prognostic marker for DSS in univariate analyses, both in the total material and in the subgroup with wide resection margins. Further, we found that VEGF-A and its corresponding receptors VEGFR-1 and -2, showed significant correlations with histological tumor grade, in accordance with previously published studies [12], [13], [17]. VEGF-A activation of its corresponding receptors, VEGFR-1 and -2, is known to be the major angiogenic pathway [8]. The close correlation between these markers and histological grade suggests that they play a role in the development of many of the non-GIST STSs, either through angiogenesis or other stroma-associated mechanisms.

Antibodies targeting the VEGF/VEGFR systems are readily available, and clinical trials with such agents have been initiated in several cancer types [28]. However, proper criteria for selecting patients to treatment with these drugs are still lacking [28]. For the employment of antiangiogenic drugs, side effects have to be carefully weighed against efficacy, especially for patients with intermediate to good prognosis. Hence, enhanced knowledge about these molecules and their impact on different types of cancer is pivotal.

As our data are prognostic and not mechanistic we cannot conclude on which pathways are operative in non-GIST STSs expressing VEGFs and VEGFRs. Nevertheless, it can be deduced that VEGFs and VEGFRs play critical roles in sarcoma progression and prognosis. But whether angiogenic ligands and receptors may have predictive effects with respect to therapy remains unanswered. The mechanistic impacts of angiogenesis, lymphangiogenesis, autocrine versus paracrine pathways as well as the relevance of constitutively activated receptors have to be further clarified. Consequently, future translational research in this field is needed.
